# Cognitive and Affective-Emotional Factors in Math Achievement: The Mediating Role of Intelligence

**DOI:** 10.3390/jintelligence14020025

**Published:** 2026-02-04

**Authors:** Yoshifumi Ikeda, Lorenzo Esposito, Yosuke Kita, Yuhei Oi, Riko Takagi, Kent Suzuki, Irene Cristina Mammarella, Sara Caviola, Silvia Lanfranchi, Francesca Pulina, David Giofrè

**Affiliations:** 1 Department of Special Needs Education, Tokyo Gakugei University, Tokyo 184-8501, Japan; yosifumi@u-gakugei.ac.jp; 2 Department of Education (Disfor), University of Genoa, 16128 Genoa, Italy; 3 Department of Psychology, Faculty of Letters, Keio University, Tokyo 108-8345, Japan; yosuke.kita@keio.jp; 4 Faculty of Liberal Arts and Sciences, Chukyo University, Aichi 470-0393, Japan; y-oi@lets.chukyo-u.ac.jp; 5 Graduate School of Education, Joetsu University of Education, Niigata 943-8512, Japan; j295261p@myjuen.jp (R.T.); j20215060c@myjuen.jp (K.S.); 6 Department of Developmental Psychology and Socialization, University of Padua, 35131 Padova, Italy; irene.mammarella@unipd.it (I.C.M.); sara.caviola@unipd.it (S.C.); silvia.lanfranchi@unipd.it (S.L.); francescapulina1@gmail.com (F.P.)

**Keywords:** math achievement, self-efficacy, anxiety, intelligence, working memory

## Abstract

In this study, we aimed to investigate the cognitive and affective-emotional factors underlying math achievement in a sample of 169 Japanese elementary school children. Using structural equation modeling, we examined the contributions of fluid and crystallized intelligence, verbal and spatial working memory, and affective-emotional variables, including general anxiety, test anxiety, math anxiety, and math self-efficacy. We found intelligence to be a strong positive predictor of math achievement, while among the affective-emotional variables, math self-efficacy emerged as the only significant predictor of math achievement. Interestingly, intelligence mediated the association between affective-emotional factors, such as math anxiety and self-efficacy, highlighting its central role in children’s math achievement. These findings underscore the strong relationship between intelligence and self-efficacy in educational contexts, suggesting that self-efficacy is closely linked to cognitive abilities to support children’s success in math. Educational implications are discussed, emphasizing the need to strengthen math self-efficacy alongside cognitive abilities.

## 1. Introduction

In our increasingly scientific and technological society, math plays a vital role in modern life and societal development. It has been recognized as relevant across a wide range of domains, spanning from employment opportunities to health conditions (Ritchie & Bates, 2013). Consequently, math achievement is considered a central aspect of educational systems worldwide (OECD, 2019), and it has been a frequent topic of psychological research. As for the mechanisms underlying achievement, studies consistently show that both domain-general and domain-specific factors (Geary et al., 2017) are involved in math achievement. From a broader perspective, research has shown that both cognitive and affective-emotional factors, including the g-factor (Esposito et al., 2025) and different types of anxiety (Caviola et al., 2022), are involved in math achievement.

### 1.1. Cognitive Factors in Math Achievement

Most of the research aimed at identifying the strength of the associations between cognitive factors and math performance is correlational. In fact, recent research has consistently found positive links between g-factor and math achievement (Esposito et al., 2025; Taub et al., 2008). In this regard, crystallized intelligence (gC), which reflects accumulated knowledge and strategies, was associated with tasks that require storing formulas, recalling facts, or applying procedural knowledge (Lozano-Blasco et al., 2022). Particularly, gC has moderate effects on math achievement across different age groups (Taub et al., 2008), particularly in tasks relying on verbal and receptive skills, such as arithmetic word problems or geometry, which involve definitions, formulas, and other verbal materials (Taub et al., 2008).

Conversely, fluid intelligence (gF), which involves reasoning and abstract problem-solving, shows a strong and positive association with tasks requiring novel approaches or conceptual understanding (Peng et al., 2019). Empirical studies showed strong positive associations between gF and math achievement (Green et al., 2017; Primi et al., 2010), with longitudinal evidence suggesting that higher gF predicts both better initial achievement and faster improvement over time (Lozano-Blasco et al., 2022; Primi et al., 2010).

Strong positive associations have also been found between working memory (WM) and math achievement across a variety of tasks, including arithmetic (Allen & Dowker, 2022; Zhang et al., 2022), geometry (Zhang et al., 2022), and overall math achievement (Caviola et al., 2022; Ji & Guo, 2023; Peng et al., 2016). Verbal working memory (WM-V) is a strong predictor of math achievement, with both direct and indirect influences (Cragg et al., 2017). In fact, it directly contributes to the construction and management of complex problem representations, and at the same time it indirectly supports domain-specific math skills, such as factual knowledge and procedural abilities. WM-V also facilitates the acquisition of formal math knowledge, as education relies heavily on verbal information such as definitions, formulas, and theorems. Extending this, meta-analytic evidence in primary school children indicated that WM-V is correlated with arithmetic tasks (Friso-van den Bos et al., 2013; Zhang et al., 2022). However, the strength of this relationship could change with age and task characteristics: younger children rely more heavily on WM-V, while its influence diminishes as math tasks become more complex and visuospatially oriented (Zhang et al., 2022). Visuospatial working memory (WM-S), which involves the temporary storage and manipulation of visual and spatial information, is a key factor that was consistently linked to math across a wide range of math fields (Allen & Dowker, 2022; Silverman & Ashkenazi, 2022; Zhang et al., 2022). It is particularly important for areas such as geometry and numeration, where mental rotation and understanding spatial relationships are essential (Silverman & Ashkenazi, 2022). Also, in procedural calculations (e.g., multi-digit subtraction), WM-S supports the correct alignment and spatial management of numbers and the maintenance of sequential steps. Its role also extends to more complex math tasks, where higher WM-S capacity is associated with better problem-solving (Allen & Dowker, 2022; Green et al., 2017).

Recent studies suggest that higher-level cognitive functions, such as the g-factor, may mediate the relationship between specific abilities (e.g., gF, gC, and WM) and math achievement (Qi et al., 2024). In this context, specific abilities are nested into higher-level intellectual abilities, which in turn influence how other individual factors relate to math achievement (Esposito et al., 2025). Overall, these findings indicate that higher-order abilities could play an important role in predicting math achievement. The effective use of these cognitive resources likely supports the acquisition of math concepts and skills and helps manage information during problem-solving tasks.

### 1.2. Affective-Emotional Factors in Math Achievement

Alongside cognitive factors such as the g-factor and WM, several other variables have been linked to math achievement. General anxiety (GA) is often considered a trait-like tendency to experience worry and tension (Eysenck et al., 2005) and shows moderate correlations with math achievement. Studies in young children indicated that higher GA is associated with lower math achievement, even when controlling for cognitive factors such as WM and g-factor, suggesting that GA alone can hinder early math learning (Cargnelutti et al., 2017). Test anxiety (TA), a context-specific form of anxiety associated with evaluative situations in educational settings, also negatively affects academic achievement, including math achievement (Caviola et al., 2022; Putwain, 2008). Among domain-specific anxieties, math anxiety (MA) is particularly relevant in educational and psychological research. It is triggered by any situation involving math content (Ashcraft & Moore, 2009) and has been shown to impair multiple aspects of math learning, including task achievement (Caviola et al., 2022; Esposito et al., 2025), engagement in math-related activities (Choe et al., 2019), and even enrollment in math-oriented courses (Daker et al., 2021). It is also worth noting that recent meta-analyses (e.g., Caviola et al., 2022; Donolato et al., 2020) suggest that not all anxiety dimensions are equally associated with math. While TA and GA can be conceptualized as domain-general, MA represents a domain-specific form that typically shows the strongest correlation with math achievement. Therefore, assessing multiple forms of anxiety allows for identifying their unique contribution to math achievement.

Alongside anxiety forms in math, a considerable line of research focused instead on the positive attitudes related to math. These are part of a multidimensional construct encompassing several aspects, such as enjoyment, value attributed to the subject and, importantly, one’s beliefs about their own competencies in math (Aiken, 1970; Putwain et al., 2017). These beliefs can be either general and stable beliefs about one’s abilities (e.g., self-concept), or specific to the tasks (Pajares & Graham, 1999). The latter notion has been termed self-efficacy (SE), highlighting the perceived ability and competence in solving a specific math task, rather than general academic achievement. Research has consistently found positive associations between SE and achievement (Ma & Kishor, 1997; Recber et al., 2018), suggesting that one’s belief in their own competence could enhance interest, motivation, and the cognitive resources allocated to solving a math task. Crucially, prior evidence suggests that self-efficacy makes one of the strongest contributions to math achievement, even when controlling for other key predictors such as intelligence and anxiety (e.g., Donolato et al., 2020). The crucial role of self-efficacy has also been examined in other subjects, showing consistent links with academic success (Honicke & Broadbent, 2016). At the same time, positive associations were found with other positive attitudes, such as self-concept (Akin & Kurbanoğlu, 2011; Arens et al., 2022), as well as negative associations with MA (Akin & Kurbanoğlu, 2011). Even though extensive research on the topic has been carried out, underlying mechanisms are still unknown, and research tried to uncover possible causal mechanisms. For instance, recent studies suggested that MA could negatively affect SE, as MA could weaken the positive belief that one has, which in turn leads to the recruitment of fewer cognitive resources required for the resolution of a specific math task (Labong et al., 2025; Palestro & Jameson, 2020). This hypothesized mechanism could be framed within Bandura’s (1977) self-efficacy theory, according to which one’s beliefs and confidence in their abilities are important factors determining the effort they invest when facing challenges.

### 1.3. Aim and Hypotheses

As outlined above, an extensive amount of research has tried to explore the contributions of cognitive and affective-emotional factors in math. While cognitive and affective-emotional factors are often investigated separately, they are not independent and likely share a large portion of variance. Consequently, analyzing them within a single comprehensive model is crucial to disentangle their unique contributions to math achievement and to determine which factors remain significant predictors when controlling for the others. This study aims to explore the complex interplay of cognitive factors (i.e., g-factor, WM) and affective-emotional variables (i.e., math SE, MA), in influencing math achievement. Based on past research, we examined to what extent these factors, assessed in second- and fifth-grade children, are associated with their math achievement. Given that previous studies have shown that cognitive and affective-emotional factors strongly influence children’s school achievement, we expected that g-factor and positive math-related affective dispositions, such as SE, would be positively associated with math achievement. Conversely, we expected negative emotional experiences, such as GA and TA, to negatively influence math achievement. Additionally, we hypothesized that MA and SE would be indirectly related to math achievement through the g-factor, with SE showing a positive association and MA a negative one.

## 2. Materials and Methods

### 2.1. Participants

The initial pool included 173 elementary school children. Four participants were excluded due to incomplete data, resulting in a final sample of 169 children (103 boys, 66 girls). The sample comprised 95 second-graders (56.31% boys) and 74 fifth-graders (60.81% boys), with a mean age of 112.92 months (approximately 9.5 years; *SD* = 18.06). Participants were recruited from three mainstream public elementary schools in Japan. Eligibility criteria required that children were native Japanese speakers and not enrolled in special education programs. The study received approval from the Ethics Committee of the University of Joetsu University of Education (protocol code 2018-62, 2021-102). Written informed consent was provided by the parents or legal guardians of all participating children, while children provided their oral assent prior to participation.

Parents or guardians provided written informed consent for 99 second-graders (39.9% of the initial pool of 248) and 74 fifth-graders (27.7% of 267). Due to incomplete data for 4 children, the final sample for second-grade comprised 95 students.

The present study is part of a broader research project on cognitive, emotional, and academic development in childhood. Some results based on this dataset have been published previously (Ikeda et al., 2023; Ikeda et al., 2025); however, none of the analyses or findings reported in this paper have been presented or disseminated elsewhere.

### 2.2. Materials

#### 2.2.1. Math Achievement Test

Children’s math abilities were assessed using the math section of the Kyokenshiki Criterion-Referenced Test-II (Tatsuno & Kitao, 2018), a widely recognized standardized test in Japan. This subtest comprises various types of items: multiple-choice, fill-in-the-blank, short-answer, and drawing problems, which evaluate skills such as number sense, arithmetic facts, computational procedures, problem-solving strategies, and understanding of shapes and measurements. Achievement is summarized across three main domains: (1) math reasoning, (2) quantitative and spatial skills, and (3) knowledge and comprehension of quantitative and spatial concepts. These areas reflect the official national curriculum guidelines for Japanese primary schools.

Because testing occurred early in the school year, each child was administered the version designed for the previous grade level. For example, second-grade children completed the first-grade version, while fifth-graders were given the fourth-grade version. This approach ensured that all participants had already been taught the relevant content. The test was conducted under a 40-min time limit and scored using the official scoring procedures. Individual scores were standardized, with higher values indicating stronger math achievement. Internal consistency was estimated using a model-based reliability index derived from confirmatory factor analysis, which showed good reliability (Cronbach’s α = 0.81).

#### 2.2.2. Intelligence

##### Crystallized Intelligence

Two subtests from the Japanese adaptation of the WISC-IV (Wechsler & Japanese WISC-IV Publication Committee, 2010) were used to assess gC: Vocabulary and Similarities. In the Vocabulary task, children were asked to define a series of orally presented words. This subtest assesses expressive vocabulary, verbal concept formation, and general verbal knowledge. Test–retest reliability for this subtest has been reported as *r* = 0.80. In the Similarities task, children were presented with pairs of words (e.g., poet and painter) and asked to explain how the two are alike. This subtest evaluates verbal abstract reasoning and the ability to identify conceptual relationships (test–retest reliability, *r* = 0.85).

##### Fluid Intelligence

The Japanese version of the Cattell Culture Fair Intelligence Test Scale 2 (Cattell & Cattell, 1960) was used to assess gF. The test consists of two forms, A and B. Each form includes four timed subtests of nonverbal fluid reasoning (series, classifications, matrices, and topology) with items of increasing difficulty within each subtest. We calculated two scores from the sum of correct answers for the form A and form B separately (test–retest reliability, *r* = 0.84).

#### 2.2.3. Working Memory

##### Verbal Working Memory

WM-V was assessed using three tasks: the Number Span Task (NST), the Word Span Task (WST), and the Listening Span Task (LST). In the Number Span Task, children listened to sequences of digits and were asked to recall them aloud in the same (forward) order. Span lengths ranged from two to eight digits, with two trials per length. Internal consistency in the present sample was high (*α* = 0.89). The Word Span Task followed the same structure, using sequences of familiar Japanese nouns instead of digits. Words were presented auditorily, and participants repeated them in the order they were heard. Reliability for this task was also high (*α* = 0.88). In the Listening Span Task (Daneman & Carpenter, 1980), children listened to sets of simple Japanese sentences, ranging from two to five per trial, with two trials per span length. After hearing each sentence, they judged whether it was semantically correct (e.g., “Bears live in the mountains” vs. “Ears eat rice”), and then, after each set, recalled the first word of each sentence in the order presented. Sentences were three words long and followed the standard Japanese Subject–Object–Verb (SOV) structure. The reliability of the Listening Span Task was high (*α* = 0.90). For the WST and LST, the target word stimuli were drawn from the Textbook Vocabulary Corpus (Tanaka et al., 2011) and met strict linguistic criteria: each noun consisted of two characters, two syllables, and two morae. To minimize phonological interference, words with identical vowel combinations (e.g., uma and kusa) were not included in the same trial sequence, and each word was used only once throughout the task.

##### Spatial Working Memory

Spatial working memory was assessed using three tasks: the Matrices Span Task (MST), the Corsi Block Task (CBT), and the Dot Matrix Task (DMT). In the Matrices Span Task, participants were presented with a sequence of briefly appearing (1 s) highlighted cells on a 5 × 5 grid. After each sequence, they were asked to recall the positions by clicking the corresponding cells in the same order using a mouse. Span lengths ranged from two to eight items, with two trials per length. Internal consistency for this task was high (*α* = 0.91). The Corsi Block Task followed the same procedure but used a visual display of nine blocks arranged irregularly on the screen instead of a grid. Children were required to reproduce the sequence of highlighted blocks in the correct order. Span lengths and trial structure mirrored the matrices task. Reliability for the Corsi task was similarly high (*α* = 0.90). In the Dot Matrix Task (Miyake et al., 2001), participants solved simple matrix-based arithmetic equations (e.g., line-based addition problems) while simultaneously encoding the position of a dot that briefly appeared in a 5 × 5 grid after each equation. After a series of two to five equation–dot pairs (with two trials at each level), children were asked to recall the sequence of dot positions in order. This dual-task condition taxed both processing and storage components of WM. Internal consistency for this task was also high (*α* = 0.90).

#### 2.2.4. Test Anxiety

Test-related anxiety was assessed using the Japanese version of the Test Anxiety Scale (Sakano, 1988), a translation of the original instrument developed by Sarason (1972). The scale comprises 16 dichotomous (yes/no) items (e.g., “I worry about doing well on tests”). Higher total scores indicate greater levels of TA. The scale’s reliability and validity have been established in both junior high school (Miura et al., 1997) and elementary school populations (Matsunuma, 2004). For the present study, minor wording adjustments were made to enhance the appropriateness of the items for younger children. Internal consistency was acceptable (*α* = 0.73).

#### 2.2.5. General Anxiety

The Japanese version of the Children’s Manifest Anxiety Scale (CMAS; Sakamoto, 1965, 1989), which is a translation of the Children’s Manifest Anxiety Scale (Castaneda et al., 1956), is a self-report questionnaire for evaluating GA in children and adolescents. It comprises 53 items (42 anxiety and 11 lie items) that utilize a yes/no response format (e.g., “I often worry about things”). Higher scores on this scale indicate a greater level of GA. Internal consistency was acceptable (*α* = 0.87).

#### 2.2.6. Math Anxiety

To assess MA, we used both the AMAS and J-MAS to create a robust latent variable, reducing measurement error specific to a single instrument (see Results, *r* = 0.83).

##### Abbreviated Math Anxiety Scale (AMAS)

Children’s levels of MA were assessed using the Japanese adaptation of the Abbreviated Math Anxiety Scale (AMAS; Ikeda et al., 2025). This 9-item instrument uses a 5-point Likert scale ranging from 1 (strongly disagree) to 5 (strongly agree) (e.g., “Thinking about an upcoming math test”). The AMAS captures two key facets of MA: anxiety associated with math achievement and evaluation (testing anxiety), and anxiety related to learning and engaging with math in everyday contexts (learning anxiety). In this study, the scale demonstrated excellent internal consistency (Cronbach’s *α* = 0.90). Higher scores indicate greater levels of MA.

##### Japanese Math Anxiety Scale (J-MAS)

The Japanese Math Anxiety Scale (J-MAS; Watanabe & Sakuma, 1998) was used as a culturally adapted measure of MA. The scale includes 18 items, each rated on a 5-point Likert scale ranging from 1 (strongly disagree) to 5 (strongly agree) (e.g., “When I cannot solve a math problem”). Items are organized into four subdomains: anxiety related to math lessons, problem-solving, interactions with the teacher, and peer comparisons during math activities. In the present study, the J-MAS demonstrated excellent internal consistency (*α* = 0.93), confirming its reliability in capturing diverse aspects of math-related anxiety in classroom contexts. Higher total scores indicate greater levels of MA.

#### 2.2.7. Math Self-Efficacy

Math SE was measured using the Japanese version of the Mathematical Self-Efficacy Scale (Matsunuma, 2004), which consists of 8 items rated on a 5-point Likert scale ranging from 1 (strongly disagree) to 5 (strongly agree) (e.g., “I am confident I can understand the math concepts”). The items were adapted from the self-efficacy subscale of the Motivated Strategies for Learning Questionnaire developed by Pintrich and De Groot (1990), which is designed to assess students’ confidence in their ability to perform math tasks and understand math content. In the current study, the scale showed excellent internal consistency (*α* = 0.93). Higher scores reflect greater levels of SE in math.

### 2.3. Analytical Approach

Analyses were performed in R (R Core Team, 2024, version 4.4), using RStudio as the IDE (RStudio Team, 2024, version 2025.9). All variables were first residualized for grade and subsequently standardized as z-scores. A series of correlation analyses was then conducted to examine the relationships among the variables. We also fitted a series of confirmatory factor analyses (CFAs) to determine the best structural representation of g-factor (see Supplementary Materials).

Subsequently, we fitted a measurement model to check that the observed variables adequately reflected the latent constructs, thereby confirming the reliability of the measures. For constructs measured with a single indicator, such as GA, TA, and SE, variances were fixed using the formula “1—Reliability” (Kline, 2023), using the internal consistency coefficients calculated in the present sample, *α* = 0.87 for GA, *α* = 0.73 for TA, and *α* = 0.93 for SE. Finally, the structural equation modeling (SEM) approach was used to explore the relationships among cognitive factors (e.g., g-factor, WM) and affective-emotional factors (e.g., GA, TA, MA, SE), and their contribution to math achievement. Specifically, we first fitted models including cognitive and affective-emotional factors separately, and then examined their combined contribution in joint models. We also examined mediation effects of MA and SE on math achievement, specifically the effect of SE via the g-factor and the effect of MA via SE and the g-factor. The lavaan package was used to perform SEM (Rosseel, 2012). Indirect effects were estimated using a Monte Carlo simulation with a significance level of α = 0.05, implemented via the semTools package (Jorgensen et al., 2025). The lavaangui package was used to plot the SEM models (Karch, 2025).

The goodness-of-fit criteria were evaluated according to guidelines proposed by Hu and Bentler (1999), who suggested a CFI (Comparative Fit Index) and NNFI (Non-normed Fit Index) greater than 0.95 as a good fit, an RMSEA (Root Mean Square Error of Approximation) less than 0.06 as an acceptable fit, and an SRMR (Standardized Root Mean Square Residual) less than 0.08 as a good fit (Hu & Bentler, 1999). The chi-square difference test (Δ*χ*^2^) was used for testing the difference between nested models. Since model comparison is not possible when models are not nested, the relative indices AIC (Akaike Information Criterion) and BIC (Bayesian Information Criterion) were used, where a decrease of 2–4 units was considered indicative of model improvement (Burnham & Anderson, 2002).

## 3. Results

### 3.1. Preliminary Analyses

Descriptive statistics, including means, standard deviations, skewness, and kurtosis for all measures were calculated and presented (see Supplementary Materials). As shown in the Table 1, correlational analyses indicated that math achievement was positively associated with cognitive abilities, including WM and gF, suggesting that stronger cognitive abilities are associated with higher math achievement. MA was negatively related to math achievement, highlighting the detrimental role of negative affective-emotional factors. In contrast, math SE showed positive associations with math outcomes, underscoring the importance of students’ confidence in their math abilities. GA and TA were only weakly associated with math achievement, indicating that math-specific affective factors could be more relevant predictors of achievement. We also checked for multicollinearity by computing the VIF for all predictors (see Supplementary Materials).

We also tested a series of structural models to examine the factorial structure of g-factor. First, a g-only model was specified, in which all cognitive indicators loaded onto a single g-factor. Second, a model including WM and g-factor as separate latent factors. Third, a full model specifying gF, gC, and both WM-V and WM-S as distinct latent factors. Finally, a hierarchical model was fitted, in which gF, gC, and WM-V and WM-S indicators loaded onto a higher-order g-factor. Among the tested models, the higher-order model showed the best overall fit, *χ*^2^(31) = 37.198, *p* = .205, CFI = 0.998, NNFI = 0.982, RMSEA = 0.034, SRMR = 0.046, AIC = 4319.736, BIC = 4394.854 (see Supplementary Materials).

### 3.2. Measurement Model

We tested a measurement model to explore the relationships among our variables and to confirm that the observed indicators adequately reflected the underlying latent constructs. We hypothesized several latent factors: math achievement; WM-V and WM-S, gF and gC, with a second-order g-factor; GA, TA, MA, and math SE. The overall fit of this model was acceptable, *χ*^2^(119) = 149.134, *p* < .05, CFI = 0.972, NNFI = 0.964, RMSEA = 0.039, SRMR = 0.050, AIC = 7633.150, BIC = 7795.905 (see Table 2). Factor loadings were interpreted following common psychometric guidelines (Stevens, 2002; Hair et al., 2019), considering loadings equal or higher than 0.35 as acceptable for this sample size. All factor loadings in our model exceeded this threshold.

### 3.3. Structural Equation Models

Having established that the model provided an adequate fit to the data, we proceeded to test several structural models aimed at identifying the strongest predictors of math achievement and whether MA and SE have indirect effects via the g-factor (see Table 3).

In Model 1, we modeled gF, gC, WM-V, and WM-S as indicators of a latent g-factor, which, in turn, predicted math achievement. The model showed good fit according to multiple indices: *χ*^2^(60) = 87.604, *p* = .012, CFI = 0.964, NNFI = 0.953, RMSEA = 0.052, SRMR = 0.053, AIC = 5528.365, BIC = 5625.391. The g-factor strongly predicted math achievement, *β* = 0.786, *p* < .001.

In Model 2, we aimed to determine which among the four affective-emotional constructs was the strongest predictor of math achievement. The model showed excellent fit: *χ*^2^(13) = 10.520, *p* = .651, CFI = 1.000, NNFI = 1.000, RMSEA = 0.000, SRMR = 0.024, AIC = 3378.048, BIC = 3450.036. Only SE was significantly associated with math achievement, *β* = 0.312, *p* = .001. Whereas GA, *β* = 0.116, *p* = .398, TA, *β* = −0.228, *p* = .110, and MA, *β* = −0.173, *p* = .071, were not significant. These results indicate that SE is the strongest predictor of math achievement when all four affective-emotional measures are included in the model.

Subsequently, in Model 3, we examined the relative contribution of all predictive factors on math achievement when included simultaneously. Model fit was good: *χ*^2^(119) = 149.134, *p* = .032, CFI = 0.972, NNFI = 0.964, RMSEA = 0.039, SRMR = 0.050, AIC = 7633.150, BIC = 7795.905. Among the predictors, only the g-factor, *β* = 0.724, *p* < .001, and SE, *β* = 0.156, *p* = .047 were significantly associated with math. In contrast, GA, *β* = −0.001, *p* = .991, TA, *β* = −0.018, *p* = .885, and MA, *β* = −0.129, *p* = .120, were not statistically significant.

In a subsequent model, we tested the direct and indirect effects of MA and SE on math achievement. Specifically, in Model 4, we considered the indirect effect of MA through SE and the g-factor, and the indirect effect of SE through the g-factor. Model fit was good: *χ*^2^(124) = 153.807, *p* = .036, CFI = 0.972, NNFI = 0.966, RMSEA = 0.038, SRMR = 0.056, AIC = 7627.823, BIC = 7774.929. Among the predictors of math, only the g-factor, *β* = 0.723, *p* < .001, was significantly associated with achievement. In contrast, GA, *β* = 0.030, *p* = .786, TA, *β* = −0.077, *p* = .506, MA, *β* = −0.147, *p* = .076, and SE, *β* = 0.152, *p* = .053, were not statistically significant. In the mediation paths, MA negatively predicted SE, *β* = −0.338, *p* < .001, and SE positively predicted the g-factor, *β* = 0.241, *p* = .011. Indirect and total effects were examined to clarify the mediating role of SE and the g-factor. The indirect effect of MA on math via SE and the g-factor was negative and significant, *β* = −0.059, 95% CI [−0.119, −0.014], whereas the indirect effect of SE via the g-factor was positive and significant, *β* = 0.174, 95% CI [0.048, 0.310]. Regarding total effects, MA showed a negative association with math, *β* = −0.206, 95% CI [−0.370, −0.045], and SE was positively associated with math, *β* = 0.327, 95% CI [0.164, 0.487]. These results indicate that SE and the g-factor could partially mediate the impact of MA on math achievement.

**Figure 1:**
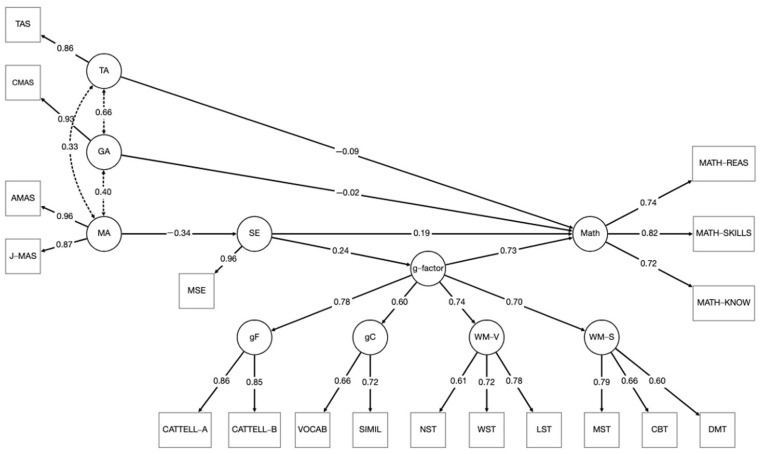
Standardized solution of Model 5. MATH-REAS = Math Reasoning (Personalized Task); MATH-SKILLS = Math Skills (Personalized Task); MATH-KNOW = Math Knowledge (Personalized Task); CATTELL-A = Cattell Culture Fair Intelligence Test, Subtest A; CATTELL-B = Cattell Culture Fair Intelligence Test, Subtest B; VOCAB = WISC Vocabulary Subtest; SIMIL = WISC Similarities Subtest; NST = Number Span Task; WST = Word Span Task; LST = Listening Span Task; MST = Matrix Span Task; CBT = Corsi Block-Tapping Task; DMT = Dot Matrix Task; CMAS = Children’s Manifest Anxiety Scale; TAS = Test Anxiety Scale; AMAS = Abbreviated Math Anxiety Scale; J-MAS = Japanese Math Anxiety Scale; MSE = Math Self-Efficacy Scale; Math = Math; g-factor = general intelligence factor; gF = fluid intelligence; gC = crystallized intelligence; WM-V = verbal working memory; WM-S = spatial working memory; GA = general anxiety; TA = test anxiety; MA = math anxiety; SE = math self-efficacy factor.

Finally, we tested a more parsimonious model in which we removed the direct effect of MA on math achievement, as its contribution, while controlling for the other factors, was not significant (see Model 3). In Model 5 (see Figure 1), fit indices were acceptable, *χ*^2^(125) = 157.071, *p* = .028, CFI = 0.970, NNFI = 0.964, RMSEA = 0.039, SRMR = 0.059, AIC = 7629.088, BIC = 7773.063. In this model the g-factor, *β* = 0.728, *p* < .001, and SE, *β* = 0.194, *p* = .012, were significantly associated with achievement, whereas GA, *β* = −0.019, *p* = .863, and TA, *β* = −0.091, *p* = .439, did not reach the significance. As for the mediation effects, MA negatively predicted SE, *β* = −0.340, *p* < .001, and SE positively predicted the g-factor, *β* = 0.241, *p* = .011. The indirect effect of MA on math via SE and g-factor was significant, *β* = −0.060, 95% CI [−0.120, −0.014], as was the indirect effect of SE via g-factor, *β* = 0.175, 95% CI [0.048, 0.310], resulting in a total significant effect of SE on math, *β* = 0.369, 95% CI [0.213, 0.519]. Although AIC and BIC values changed between the last two models, the models did not differ significantly, Δ*χ*^2^(1) = 3.26, *p* = .071, suggesting that the direct effect of MA on math was negligible.

## 4. Discussion

The present study explored the role of cognitive and affective-emotional factors in shaping math achievement in second- and fifth-grade children. By combining measures of g-factor, GA, TA, MA, and SE, we aimed to provide a comprehensive view of the cognitive and affective-emotional factors that could impact math learning. Overall, our findings underscore the interplay between cognitive abilities and affective-emotional factors, highlighting the importance of considering both domains when exploring math achievement. Interestingly, our results highlight the importance of cognitive abilities when simultaneously considering SE and forms of anxiety in explaining math achievement.

### 4.1. Findings on Cognitive Factors

In line with recent research on the topic (Esposito et al., 2025), we found positive relationships between cognitive abilities, such as WM and g-factor measures. As for the g-factor, our results showed positive associations with measures of gF and gC, as they could support the ability to apply abstract rules, use stored knowledge which in turn favors math learning (Cowan, 2017). In math, gF is particularly important for tasks that require conceptual understanding and problem-solving in new situations, while gC is crucial for tasks that rely on learned notions, such as arithmetic facts and formulae application. These findings align with previous research showing positive associations between both gF and gC and children’s math achievement (Peng et al., 2019).

Specifically, our correlation analysis revealed moderate positive associations between all math subtests and our measures of both WM-V and WM-S. These findings align with previous research showing that children with higher WM resources tend to perform better in math (Gaye et al., 2024; Peng et al., 2016). In particular, WM could facilitate the temporary storage and manipulation of important information during problem-solving. Specifically, WM-V could support tasks that require the storage and manipulation of numerical and verbal information, such as arithmetic word problems, while WM-S is crucial for tasks involving geometric reasoning and mental rotation. These findings are consistent with previous studies highlighting the roles of WM in math (Friso-van den Bos et al., 2013; Allen & Dowker, 2022).

Our structural models extended previous findings, highlighting the pivotal role of g-factor in shaping math achievement. The g-factor, capturing gF, gC, WM-V, and WM-S as indicators of higher-order abilities, strongly predicted math achievement, beyond the contribution of affective-emotional factors (Esposito et al., 2025). In other words, the contribution of the g-factor suggests that math achievement heavily depends on general cognitive capacity rather than on specific skills. These results are consistent with prior research showing that general cognitive abilities are stronger predictors of math achievement than individual cognitive abilities alone (Qi et al., 2024; Green et al., 2017). In line with these results, meta-analytic evidence suggested that general cognitive abilities are robust across math domains (Friso-van den Bos et al., 2013), further highlighting the central role of the g-factor. Moreover, our findings suggest that g-factor could mediate not only the contribution of lower-order cognitive abilities, such as WM and individual g-factor components (e.g., gC and gF), but also the relationship of affective-emotional factors, including MA and math SE, on math achievement. This mediating role indicates that general cognitive ability organizes and integrates both cognitive and affective resources to support effective problem-solving and learning in math (Esposito et al., 2025; Qi et al., 2024). Taken together, these findings highlight that cognitive abilities, as represented by a general g-factor factor, are essential predictors of math achievement in children, providing a robust foundation for the influence of other factors.

### 4.2. Findings on Affective-Emotional Factors

Our results highlight the important role of affective-emotional factors in math achievement. Correlations showed that MA was negatively associated with math achievement, while math SE showed a strong positive association. In contrast, GA and TA were only weakly associated with math achievement, suggesting that domain-specific factors have a stronger impact on math achievement than more general emotional traits (Demedts et al., 2022).

Our structural models extended previous findings, highlighting important nuances of affective-emotional factors in shaping math achievement. For instance, SE was the only significant predictor of math achievement, when all affective-emotional variables and g-factor were included in the same model. Overall, these findings suggest that the perception of one’s own math abilities plays an important role and contributes to math achievement above and beyond anxiety measures.

We also explored indirect associations of MA and SE with mediation analyses. As regards the SE, we found a positive and indirect effect via the g-factor, indicating that higher SE could favor the engagement of cognitive resources, such as reasoning and WM, which in turn could aid math achievement. Conversely, MA showed a negative indirect association with math achievement, via SE and the g-factor. This pattern suggests that MA could have a detrimental effect on achievement by negatively impacting students’ perception of their own abilities and cognitive resources. Crucially, this result suggests that the effect of MA on achievement is indirect, that is undermining children’s confidence and beliefs. Subsequently, this weakened self-belief could prevent the full recruitment of available cognitive resources, ultimately limiting achievement (Labong et al., 2025; Palestro & Jameson, 2020).

These results strongly align with the SE theory (Bandura, 1977), which posits that individuals’ beliefs in their own abilities influence how cognitive resources are recruited during learning. Moreover, consistent with the Control-Value Theory (Pekrun, 2006), which emphasizes that achievement-related emotions and control appraisals (e.g., SE, MA) strongly affect achievement, our findings reveal a clear link between SE and math achievement through the g-factor. These results highlight the complex nature of affective and cognitive components in math achievement. While the g-factor has a stable role on achievement, affective-emotional variables, particularly SE, appear to influence how cognitive abilities are engaged during the resolution of math tasks.

Finally, the role of SE and MA should be considered within the Japanese educational context. From the earliest stages of schooling, the Japanese educational system emphasizes effort and frequent assessment, making students’ beliefs in their own abilities a crucial resource for coping with academic pressure and for sustaining engagement in math. This could explain why SE was more strongly associated with math achievement than broader forms of anxiety.

### 4.3. Future Research

Some limitations of this study should be acknowledged. First, although our final structural model demonstrated acceptable fit indices (e.g., CFI, RMSEA), the *χ*^2^ statistic was significant. While this outcome is common in samples of this size and complexity, it suggests the presence of minor unmodeled variances. In particular, the direct path from MA to math achievement was not trivial (i.e., 15). Although this effect did not reach statistical significance, its magnitude may indicate that math anxiety could exert a modest direct influence on math performance. Second, the cross-sectional design does not allow causal interpretations of the observed relationships. Longitudinal approaches could clarify the directionality of the links between cognitive and affective-emotional factors in math learning during development. Specifically, while our model places cognitive abilities as a mediator for SE, reciprocal relationships are plausible. Future longitudinal studies are needed to disentangle whether higher intelligence fosters SE or if beliefs support the expression of cognitive potential. Third, our sample was limited to second- and fifth-grade children, which limits the generalizability of our findings to other age groups. Future studies could explore the dynamic relationships among these variables in older age groups. Fourth, the use of self-report measures to assess all affective-emotional constructs (e.g., MA) may have introduced some bias related to subjective reporting. Future studies could benefit from including multi-method assessments, such as physiological indicators of anxiety, to better understand and extend these findings. Moreover, the study was conducted in a specific cultural context (i.e., Japan). Given the specific features of the Japanese educational system, often characterized by high academic standards and distinct cultural values regarding effort and achievement, our findings might not be immediately generalizable to other Western educational contexts.

### 4.4. Practical Implications

These findings have important implications for educational practices. In fact, they show that alongside cognitive abilities such as g-factor and WM, other factors such as MA and SE are central for accomplishments in math domains (Clemente et al., 2024; Zakariya, 2022). Evidence suggests that strategies aimed at increasing math SE are most effective when they address multiple sources, such as providing students with mastery experiences, opportunities to observe peers successfully completing tasks, constructive feedback and encouragement, and support for positive affective states. Furthermore, our results suggest that interventions should be tailored to the students’ cognitive resources. Since intelligence plays a central mediating role, simply reducing anxiety might not be enough if the task exceeds the child’s cognitive capacity. Teachers should therefore implement scaffolding techniques that break down complex problems into manageable steps. This approach may favor students to increase experiences, thereby breaking the cycle of anxiety and poor performance. We focused on elementary school children to understand the early emergence of anxiety. While the role of anxiety is well-established in older students, our findings suggest that these emotional factors begin to exert a considerable influence on math achievement at very early stages of schooling, suggesting that interventions should start earlier than typically assumed.

## 5. Conclusions

This study highlights the joint contribution of cognitive and affective-emotional factors to math achievement in children. The higher-order g-factor, encompassing WM, gF, and gC, was a strong predictor of math achievement. Interestingly, math SE emerged as the strongest predictor among affective-emotional factors, associated with achievement directly and indirectly via the g-factor. Conversely, MA had an indirect effect on achievement through its impact on SE and g-factor. Therefore, alongside cognitive ability, students’ beliefs and confidence in their skills play an important role in their achievement. The higher-order g-factor played a central mediating role, linking both math SE and MA on children’s math achievement. Overall, these findings emphasize that math achievement relies on the interplay between cognitive resources and affective-motivational processes.

## Figures and Tables

**Table 1:** Pearson’s correlations.

	1	2	3	4	5	6	7	8	9	10	11	12	13	14	15	16	17
1. MATH-REAS	1																
2. MATH-SKILLS	0.60 **	1															
3. MATH-KNOW	0.51 **	0.62 **	1														
4. CATTELL-A	0.42 **	0.47 **	0.44 **	1													
5. CATTELL-B	0.39 **	0.45 **	0.42 **	0.74 **	1												
6. VOCAB	0.30 **	0.26 **	0.37 **	0.24 **	0.16 *	1											
7. SIMIL	0.22 **	0.30 **	0.23 **	0.35 **	0.26 **	0.47 **	1										
8. NST	0.26 **	0.27 **	0.35 **	0.23 **	0.26 **	0.21 **	0.19 *	1									
9. WST	0.14	0.25 **	0.25 **	0.25 **	0.29 **	0.21 **	0.21 **	0.53 **	1								
10. LST	0.28 **	0.32 **	0.39 **	0.41 **	0.44 **	0.32 **	0.33 **	0.42 **	0.56 **	1							
11. MST	0.32 **	0.29 **	0.31 **	0.33 **	0.36 **	0.15	0.17 *	0.25 **	0.38 **	0.38 **	1						
12. CBT	0.26 **	0.37 **	0.26 **	0.34 **	0.34 **	0.15	0.14	0.25 **	0.29 **	0.37 **	0.51 **	1					
13. DMT	0.28 **	0.24 **	0.15	0.27 **	0.27 **	0.06	0.15	0.16 *	0.21 **	0.36 **	0.50 **	0.36 **	1				
14. CMAS	−0.17 *	−0.09	−0.12	−0.08	−0.11	−0.02	0.03	0.13	−0.10	−0.03	−0.08	−0.07	−0.03	1			
15. TAS	−0.18 *	−0.18 *	−0.10	−0.19 *	−0.14	−0.05	−0.06	0.03	−0.09	−0.07	−0.13	−0.10	−0.07	0.53 **	1		
16. AMAS	−0.25 **	−0.25 **	−0.18 *	−0.12	−0.18 *	−0.11	−0.03	−0.04	−0.03	−0.06	−0.10	−0.09	−0.14	0.36 **	0.27 **	1	
17. J-MAS	−0.18 *	−0.23 **	−0.15 *	−0.05	−0.05	−0.03	−0.02	−0.04	−0.04	0.02	−0.06	−0.05	−0.05	0.34 **	0.23 **	0.83 **	1
18. MSE	0.33 **	0.29 **	0.22 **	0.18 *	0.14	0.25 **	0.18 *	0.10	0.08	0.10	0.10	−0.02	0.10	−0.16 *	−0.08	−0.32 **	−0.27 **

*Note*. MATH-REAS = Math Reasoning (Personalized Task); MATH-SKILLS = Math Skills (Personalized Task); MATH-KNOW = Math Knowledge (Personalized Task); CATTELL-A = Cattell Culture Fair Intelligence Test, Subtest A; CATTELL-B = Cattell Culture Fair Intelligence Test, Subtest B; VOCAB = WISC Vocabulary Subtest; SIMIL = WISC Similarities Subtest; NST = Number Span Task; WST = Word Span Task; LST = Listening Span Task; MST = Matrix Span Task; CBT = Corsi Block-Tapping Task; DMT = Dot Matrix Task; CMAS = Children’s Manifest Anxiety Scale; TAS = Test Anxiety Scale; AMAS = Abbreviated Math Anxiety Scale; J-MAS = Japanese Math Anxiety Scale; MSE = Math Self-Efficacy Scale * *p* < .05 ** *p* < .01.

**Table 2:** Factors’ loadings (top) and latent covariances (bottom) of the measurement model.

Variable	Math	g-Factor	gF	gC	WM-V	WM-S	GA	TA	MA	SE
MATH-REAS	0.725									
MATH-SKILLS	0.829									
MATH-KNOW	0.738									
gF		0.787								
gC		0.596								
WM-V		0.728								
WM-S		0.695								
CATTELL-A			0.870							
CATTELL-B			0.846							
VOCAB				0.655						
SIMIL				0.725						
NST					0.610					
WST					0.721					
LST					0.782					
MST						0.790				
CBT						0.655				
DMT						0.600				
CMAS							0.932			
TAS								0.859		
AMAS									0.958	
J-MAS									0.871	
MSE										0.964
Math	1									
g-factor	0.786	1								
gF	0.619	0.787	1							
gC	0.469	0.596	0.469	1						
WM-V	0.572	0.728	0.573	0.434	1					
WM-S	0.547	0.695	0.547	0.414	0.506	1				
GA	−0.165	−0.101	−0.079	−0.06	−0.073	−0.07	1			
TA	−0.237	−0.222	−0.175	−0.132	−0.162	−0.154	0.665	1		
MA	−0.306	−0.163	−0.128	−0.097	−0.119	−0.113	0.403	0.324	1	
SE	0.375	0.239	0.188	0.143	0.174	0.167	−0.174	−0.100	−0.336	1

*Note*. MATH-REAS = Math Reasoning (Personalized Task); MATH-SKILLS = Math Skills (Personalized Task); MATH-KNOW = Math Knowledge (Personalized Task); CATTELL-A = Cattell Culture Fair Intelligence Test, Subtest A; CATTELL-B = Cattell Culture Fair Intelligence Test, Subtest B; VOCAB = WISC Vocabulary Subtest; SIMIL = WISC Similarities Subtest; NST = Number Span Task; WST = Word Span Task; LST = Listening Span Task; MST = Matrix Span Task; CBT = Corsi Block-Tapping Task; DMT = Dot Matrix Task; CMAS = Children’s Manifest Anxiety Scale; TAS = Test Anxiety Scale; AMAS = Abbreviated Math Anxiety Scale; J-MAS = Japanese Math Anxiety Scale; MSE = Math Self-Efficacy Scale; Math = Math; g-factor = general intelligence factor; gF = fluid intelligence; gC = crystallized intelligence; WM-V = verbal working memory; WM-S = spatial working memory; GA = general anxiety; TA = test anxiety; MA = math anxiety; SE = math self-efficacy factor.

**Table 3:** Summary of structural equation models.

Model	*χ* ^2^	*df*	*p*(*χ*^2^)	CFI	NNFI	RMSEA	SRMR	AIC	BIC
1	87.604	60	0.012	0.964	0.953	0.052	0.053	5528.365	5625.391
2	10.520	13	0.651	1.000	1.000	0.000 ^a^	0.024	3378.048	3450.036
3	149.134	119	0.032	0.972	0.964	0.039	0.050	7633.150	7795.905
4	153.807	124	0.036	0.972	0.966	0.038	0.056	7627.823	7774.929
5	157.071	125	0.028	0.970	0.964	0.039	0.059	7629.088	7773.063

*Note*. ^a^ Values reported as 0.000 are due to rounding to three decimal places.

## Data Availability

The data presented in this study are available on request from the corresponding author due to the sensitive nature of the research, as the participants did not give written consent for their data to be shared publicly.

## Supplementary Materials

The following supporting information can be downloaded at: https://www.mdpi.com/article/10.3390/jintelligence14020025/s1, Table S1: Descriptive statistics; Table S2: VIFs for all predictors; Table S3: Model comparison of intelligence.

## References

[B1-jintelligence-14-00025] Aiken L. R. (1970). Attitudes toward mathematics. Review of Educational Research.

[B2-jintelligence-14-00025] Akin A., Kurbanoğlu İ. (2011). The relationships between math anxiety, math attitudes, and self-efficacy: A structural equation model. Studia Psychologica.

[B3-jintelligence-14-00025] Allen L., Dowker A. (2022). Spatial working memory counts: Evidence for a specific association between visuo-spatial working memory and arithmetic in children. International Electronic Journal of Elementary Education.

[B4-jintelligence-14-00025] Arens A. K., Frenzel A. C., Goetz T. (2022). Self-concept and self-efficacy in math: Longitudinal interrelations and reciprocal linkages with achievement. The Journal of Experimental Education.

[B5-jintelligence-14-00025] Ashcraft M. H., Moore A. M. (2009). Mathematics anxiety and the affective drop in performance. Journal of Psychoeducational Assessment.

[B6-jintelligence-14-00025] Bandura A. (1977). Self-efficacy: Toward a unifying theory of behavioral change. Psychological Review.

[B7-jintelligence-14-00025] Burnham K. P., Anderson D. R. (2002). Model selection and multimodel inference: A practical information-theoretic approach.

[B8-jintelligence-14-00025] Cargnelutti E., Tomasetto C., Passolunghi M. C. (2017). The interplay between affective and cognitive factors in shaping early proficiency in mathematics. Trends in Neuroscience and Education.

[B9-jintelligence-14-00025] Castaneda A., McCandless B. R., Palermo D. S. (1956). The children’s form of the manifest anxiety scale. Child Development.

[B10-jintelligence-14-00025] Cattell R. B., Cattell A. K. S. (1960). Handbook for the individual or group Culture Fair Intelligence Test.

[B11-jintelligence-14-00025] Caviola S., Toffalini E., Giofrè D. (2022). Math performance and academic anxiety forms, from socio-demographic to cognitive aspects: A meta-analysis on 906,311 participants. Educational Psychology Review.

[B12-jintelligence-14-00025] Choe K. W., Jenifer J. B., Rozek C. S., Berman M. G., Beilock S. L. (2019). Calculated avoidance: Math anxiety predicts math avoidance in effort-based decision-making. Science Advances.

[B13-jintelligence-14-00025] Clemente J., Kilag O. K., Ypon A., Groenewald E., Groenewald C. A., Ubay R. (2024). Enhancing mathematics self-efficacy: Intervention strategies and effectiveness–A systematic review. International Multidisciplinary Journal of Research for Innovation, Sustainability, and Excellence (IMJRISE).

[B14-jintelligence-14-00025] Cowan N. (2017). The many faces of working memory and short-term storage. Psychonomic Bulletin & Review.

[B15-jintelligence-14-00025] Cragg L., Keeble S., Richardson S., Roome H. E., Gilmore C. (2017). Direct and indirect influences of executive functions on mathematics achievement. Cognition.

[B16-jintelligence-14-00025] Daker R. J., Gattas S. U., Sokolowski H. M., Green A. E., Lyons I. M. (2021). First-year students’ math anxiety predicts STEM avoidance and underperformance throughout university, independently of math ability. NPJ Science of Learning.

[B17-jintelligence-14-00025] Daneman M., Carpenter P. A. (1980). Individual differences in working memory and reading. Journal of Verbal Learning and Verbal Behavior.

[B18-jintelligence-14-00025] Demedts F., Reynvoet B., Sasanguie D., Depaepe F. (2022). Unraveling the role of math anxiety in students’ math performance. Frontiers in Psychology.

[B19-jintelligence-14-00025] Donolato E., Toffalini E., Giofrè D., Caviola S., Mammarella I. C. (2020). Going beyond mathematics anxiety in primary and middle school students: The role of ego-resiliency in mathematics. Mind, Brain, and Education.

[B20-jintelligence-14-00025] Esposito L., Tonizzi I., Usai M. C., Giofrè D. (2025). Understanding the role of cognitive abilities and math anxiety in adolescent math achievement. Journal of Intelligence.

[B21-jintelligence-14-00025] Eysenck M. W., Payne S., Derakshan N. (2005). Trait anxiety, visuospatial processing, and working memory. Cognition and Emotion.

[B22-jintelligence-14-00025] Friso-van den Bos I., van der Ven S., Kroesbergen E., van Luit J. E. H. (2013). Working memory and mathematics in primary school children: A meta-analysis. Educational Research Review.

[B23-jintelligence-14-00025] Gaye F., Groves N. B., Chan E. S. M., Cole A. M., Jaisle E. M., Soto E. F., Kofler M. J. (2024). Working memory and math skills in children with and without ADHD. Neuropsychology.

[B24-jintelligence-14-00025] Geary D. C., Nicholas A., Li Y., Sun J. (2017). Developmental change in the influence of domain-general abilities and domain-specific knowledge on mathematics achievement: An eight-year longitudinal study. Journal of Educational Psychology.

[B25-jintelligence-14-00025] Green C. T., Bunge S. A., Briones Chiongbian V., Barrow M., Ferrer E. (2017). Fluid reasoning predicts future mathematical performance among children and adolescents. Journal of Experimental Child Psychology.

[B26-jintelligence-14-00025] Hair J. F., Black W. C., Babin B. J., Anderson R. E. (2019). Multivariate data analysis.

[B27-jintelligence-14-00025] Honicke T., Broadbent J. (2016). The influence of academic self-efficacy on academic performance: A systematic review. Educational Research Review.

[B28-jintelligence-14-00025] Hu L.-T., Bentler P. M. (1999). Cutoff criteria for fit indexes in covariance structure analysis: Conventional criteria versus new alternatives. Structural Equation Modeling.

[B30-jintelligence-14-00025] Ikeda Y., Kita Y., Oi Y., Okuzumi H., Lanfranchi S., Pulina F., Mammarella I. C., Allen K., Giofrè D. (2023). The structure of working memory and its relationship with intelligence in Japanese children. Journal of Intelligence.

[B29-jintelligence-14-00025] Ikeda Y., Kita Y., Takagi R., Suzuki K., Mammarella I. C., Caviola S., Lanfranchi S., Pulina F., Giofrè D. (2025). The Abbreviated Math Anxiety Scale (AMAS): Applicability and utility in a sample of Japanese elementary school children. International Journal of Psychology.

[B31-jintelligence-14-00025] Ji Z., Guo K. (2023). The association between working memory and mathematical problem solving: A three-level meta-analysis. Frontiers in Psychology.

[B32-jintelligence-14-00025] Jorgensen T. D., Pornprasertmanit S., Schoemann A. M., Rosseel Y. (2025). semTools: Useful tools for structural equation modeling *(R package version 0.5-6) [Computer software]*.

[B33-jintelligence-14-00025] Karch J. D. (2025). Lavaangui: A web-based graphical interface for specifying lavaan models by drawing path diagrams. Structural Equation Modeling: A Multidisciplinary Journal.

[B34-jintelligence-14-00025] Kline R. B. (2023). Principles and practice of structural equation modeling.

[B35-jintelligence-14-00025] Labong A. L., Laum H. D., Yurango C. P. (2025). The mediating analysis of self-efficacy on the relationship between anxiety and academic performance in mathematics of grade 9 students. Asian Journal of Education and Social Studies.

[B36-jintelligence-14-00025] Lozano-Blasco R., Quílez-Robres A., Usán P., Salavera C., Casanovas-López R. (2022). Types of intelligence and academic performance: A systematic review and meta-analysis. Journal of Intelligence.

[B37-jintelligence-14-00025] Ma X., Kishor N. (1997). Assessing the relationship between attitude toward mathematics and achievement in mathematics: A meta-analysis. Journal for Research in Mathematics Education.

[B38-jintelligence-14-00025] Matsunuma M. (2004). Test anxiety, self-efficacy, self-regulated learning, and test performance: 4th grade students and an arithmetic test. The Japanese Journal of Educational Psychology.

[B39-jintelligence-14-00025] Miura M., Shimada H., Sakano Y. (1997). Successive changes of test anxiety in junior high school students: From the viewpoint of psychological stress. The Japanese Journal of Educational Psychology.

[B40-jintelligence-14-00025] Miyake A., Friedman N. P., Rettinger D. A., Shah P., Hegarty M. (2001). How are visuospatial working memory, executive functioning, and spatial abilities related? A latent-variable analysis. Journal of Experimental Psychology: General.

[B41-jintelligence-14-00025] OECD (2019). PISA 2018 results (Volume I): What students know and can do.

[B42-jintelligence-14-00025] Pajares F., Graham L. (1999). Self-efficacy, motivation constructs, and mathematics performance of entering middle school students. Contemporary Educational Psychology.

[B43-jintelligence-14-00025] Palestro J., Jameson M. (2020). Math self-efficacy, not emotional self-efficacy, mediates the math anxiety-performance relationship in undergraduate students. Cognition, Brain, Behavior. An Interdisciplinary Journal.

[B44-jintelligence-14-00025] Pekrun R. (2006). The control-value theory of achievement emotions: Assumptions, corollaries, and implications for educational research and practice. Educational Psychology Review.

[B45-jintelligence-14-00025] Peng P., Namkung J., Barnes M., Sun C. (2016). A meta-analysis of mathematics and working memory: Moderating effects of working memory domain, type of mathematics skill, and sample characteristics. Journal of Educational Psychology.

[B46-jintelligence-14-00025] Peng P., Wang T., Wang C., Lin X. (2019). A meta-analysis on the relation between fluid intelligence and reading/mathematics: Effects of tasks, age, and social economics status. Psychological Bulletin.

[B47-jintelligence-14-00025] Pintrich P. R., De Groot E. V. (1990). Motivational and self-regulated learning components of classroom academic performance. Journal of Educational Psychology.

[B48-jintelligence-14-00025] Primi R., Ferrão M. E., Almeida L. S. (2010). Fluid intelligence as a predictor of learning: A longitudinal multilevel approach applied to math. Learning and Individual Differences.

[B50-jintelligence-14-00025] Putwain D. W. (2008). Test anxiety and GCSE performance: The effect of gender and socio-economic background. Educational Psychology in Practice.

[B49-jintelligence-14-00025] Putwain D. W., Becker S., Symes W., Pekrun R. (2017). Reciprocal relations between students’ academic enjoyment, boredom, and achievement over time. Learning and Instruction.

[B51-jintelligence-14-00025] Qi Y., Chen Y., Yu X., Yang X., He X., Ma X. (2024). The relationships among working memory, inhibitory control, and mathematical skills in primary school children: Analogical reasoning matters. Cognitive Development.

[B52-jintelligence-14-00025] R Core Team (2024). R: A language and environment for statistical computing.

[B53-jintelligence-14-00025] Recber S., Isiksal M., Koç Y. (2018). Investigating self-efficacy, anxiety, attitudes and mathematics achievement regarding gender and school type. Anales de Psicología/Annals of Psychology.

[B54-jintelligence-14-00025] Ritchie S. J., Bates T. C. (2013). Enduring links from childhood mathematics and reading achievement to adult socioeconomic status. Psychological Science.

[B55-jintelligence-14-00025] Rosseel Y. (2012). Lavaan: An R package for structural equation modeling. Journal of Statistical Software.

[B56-jintelligence-14-00025] RStudio Team (2024). RStudio: Integrated development environment for R.

[B57-jintelligence-14-00025] Sakamoto T. (1965). The study on children’s manifest anxiety scale. Research Reports of Kochi University, Humanities.

[B58-jintelligence-14-00025] Sakamoto T. (1989). Manual of the Japanese version of CMAS.

[B59-jintelligence-14-00025] Sakano Y. (1988). Successive changes of test anxiety in high school students. Waseda Journal of Human Sciences.

[B60-jintelligence-14-00025] Sarason I. G., Spielberger C. D. (1972). Experimental approaches to test anxiety: Attention and the uses of information. Anxiety: Current trends in theory and research.

[B61-jintelligence-14-00025] Silverman S., Ashkenazi S. (2022). The unique role of spatial working memory for mathematics performance. Journal of Numerical Cognition.

[B62-jintelligence-14-00025] Stevens J. P. (2002). Applied multivariate statistics for the social sciences.

[B63-jintelligence-14-00025] Tanaka M., Aizawa M., Saito T., Tanahashi H., Kondo A., Kawauchi A., Suzuki K., Hirayama Y. (2011). Development and utilization of vocabulary lists, kanji lists, and other resources based on corpora for effective language policy *(Report No. LR-CCG-10-07)*.

[B64-jintelligence-14-00025] Tatsuno C., Kitao N. (2018). The manual for Kyokenshiki criterion-referenced test (CRT–II).

[B65-jintelligence-14-00025] Taub G. E., Keith T. Z., Floyd R. G., McGrew K. S. (2008). Effects of general and broad cognitive abilities on mathematics achievement. School Psychology Quarterly.

[B66-jintelligence-14-00025] Watanabe R., Sakuma T. (1998). A study on structures of children’s arithmetic anxiety and methods of teacher’s supports: Examination from social support. The Japanese Journal of Educational Psychology.

[B67-jintelligence-14-00025] Wechsler D., Japanese WISC-IV Publication Committee (2010). Japanese version of the Wechsler intelligence scale for children-fourth edition.

[B68-jintelligence-14-00025] Zakariya Y. F. (2022). Improving students’ mathematics self-efficacy: A systematic review of intervention studies. Frontiers in Psychology.

[B69-jintelligence-14-00025] Zhang Y., Tolmie A., Gordon R. (2022). The relationship between working memory and arithmetic in primary school children: A meta-analysis. Brain Sciences.

