# Supplementary material for: Cognitive and Affective-Emotional Factors in Math Achievement: The Mediating Role of Intelligence
Source: J Intell. 2026 Feb 4;14(2):25. doi: 10.3390/jintelligence14020025 (PMC12941965; doi:10.3390/jintelligence14020025)
Supplement: Supplementary file 1 [file jintelligence-14-00025-s001.zip › jintelligence-4078252-supplementary.pdf]

Cognitive and Affective-Emotional Factors in Math Achievement:

The Mediating Role of Intelligence

Supplementary Materials

Descriptive statistics and preliminary checks

Descriptive statistics, including M, SD, skewness, and kurtosis are presented in Table S1. VIF was also calculated for each variable

Table S1

Descriptive statistics

	M	*SD*	Skewness	Kurtosis
1. MATH-REAS	12.13	3.94	-0.62	0.04
2. MATH-SKILLS	17.70	4.66	-0.75	0.36
3. MATH-KNOW	10.12	2.24	-1.16	1.07
4. CATTELL-A	26.79	6.24	-0.27	-0.19
5. CATTELL-B	27.15	6.63	-0.59	-0.16
6. VOCAB	20.47	7.41	0.91	0.50
7. SIMIL	15.86	5.54	0.34	-0.72
8. NST	32.18	5.73	-0.43	-0.53
9. WST	26.43	8.10	0.61	0.81
10. LST	12.37	6.11	0.15	-0.75
11. MST	43.92	14.29	-0.05	-0.18
12. CBT	40.83	10.51	-0.33	-0.14
13. DMT	14.56	6.09	-0.04	-0.98
14. CMAS	16.26	8.52	0.33	-0.53
15. TAS	5.59	3.18	0.49	-0.21
16. AMAS	20.16	9.43	0.73	-0.33
17. J-MAS	46.35	17.07	0.23	-0.62
18. MSE	27.40	9.12	-0.57	-0.60

Note. MATH-REAS = Math Reasoning (Personalized Task); MATH-SKILLS = Math Skills (Personalized Task); MATH-KNOW = Math Knowledge (Personalized Task); CATTELL-A = Cattell Culture Fair Intelligence Test, Subtest A; CATTELL-B = Cattell Culture Fair Intelligence Test, Subtest B; VOCAB = WISC Vocabulary Subtest; SIMIL = WISC Similarities Subtest; NST = Number Span Task; WST = Word Span Task; LST = Listening Span Task; MST = Matrix Span Task; CBT = Corsi Block-Tapping Task; DMT = Dot Matrix Task; CMAS = Children's Manifest Anxiety Scale; TAS = Test Anxiety Scale; AMAS = Abbreviated Math Anxiety Scale; J-MAS = Japanese Math Anxiety Scale; MSE = Math Self-Efficacy Scale

Multicollinearity and VIF

To ensure that the inclusion of multiple variables did not introduce multicollinearity, we calculated the VIF. As shown in Table S2, all VIF values were below the conservative cutoff of 5, confirming that the predictors provide unique information. All VIF values were below the commonly used threshold of 10, which is often taken as indicative of serious or harmful multicollinearity (e.g., Marquardt, 1970; Neter et al., 1989; Hair et al., 1995). Nevertheless, relatively higher VIF values were observed for the two math anxiety measures (AMAS and J-MAS) and for some intelligence related indicators, reflecting substantial shared variance among conceptually overlapping constructs. To address this issue, math anxiety was modeled as a single latent factor using both scales as indicators, and cognitive measures were modeled within a higher order g-factor framework using structural equation modeling, thereby reducing potential multicollinearity.

Table S2

VIFs for all predictors

	VIF
CATTELL-A	2.47
CATTELL-B	2.50
VOCAB	1.45
SIMIL	1.45
NST	1.56
WST	1.86
LST	1.96
MST	1.77
CBT	1.52
DMT	1.47
CMAS	1.63
TAS	1.47
AMAS	3.85
J-MAS	3.58
MSE	1.23

Note. CATTELL-A = Cattell Culture Fair Intelligence Test, Subtest A; CATTELL-B = Cattell Culture Fair Intelligence Test, Subtest B; VOCAB = WISC Vocabulary Subtest; SIMIL = WISC Similarities Subtest; NST = Number Span Task; WST = Word Span Task; LST = Listening Span Task; MST = Matrix Span Task; CBT = Corsi Block-Tapping Task; DMT = Dot Matrix Task; CMAS = Children's Manifest Anxiety Scale; TAS = Test Anxiety Scale; AMAS = Abbreviated Math Anxiety Scale; J-MAS = Japanese Math Anxiety Scale; MSE = Math Self-Efficacy Scale; AMAS and J-MAS measures show the highest VIF values, reflecting substantial shared variance among conceptually overlapping constructs.

Factor Structure of Intelligence

Several models to test the factor structure of intelligence were tested (Table S3). In model 1, a g-only model was specified, in which all cognitive indicators loaded on a single g-factor. In Model 2, a model including WM and g-factor as separate latent factors. In Model 3, a full model specified both Gf and Gc, and both WM-V and WM-S as distinct latent factors. Finally, in Model 4, a hierarchical model was fitted, in which Gf, Gc, WM-V, and WM-S indicators loaded onto a higher-order g-factor.

Table S3

Model comparison of intelligence

Model	χ^2	*df*	p (χ^2)	CFI	NNFI	RMSEA	SRMR	AIC	BIC
1	180.457	35	< .001	.713	.631	0.157	0.095	4454.995	4517.593
2	114.075	34	< .001	.842	.791	0.118	0.086	4390.613	4456.341
3	32.522	29	= .297	.993	.989	0.027	0.039	4319.060	4400.437
4	37.198	31	= .205	.988	.982	0.034	0.046	4319.736	4394.854
